# Doctors’ knowledge, attitudes and practices of palliative care in two South African districts

**DOI:** 10.4102/phcfm.v16i1.4503

**Published:** 2024-09-30

**Authors:** Tessa A. McMillan, Lauren Hutton, Louis Jenkins

**Affiliations:** 1 Department of Family and Emergency Medicine, Faculty of Medicine and Health Sciences, Stellenbosch University, Cape Town, South Africa; 2 PHC Directorate; Family, Community and Emergency Care, Faculty of Health Sciences, University of Cape Town, Cape Town, South Africa; 3 Department of Family and Emergency Medicine, George Hospital, Western Cape Department of Health, George, South Africa

**Keywords:** palliative care, knowledge, attitudes, practice, public sector doctors

## Abstract

**Background:**

In South Africa, most palliative care takes place in health districts as part of home-based care provided by nongovernment organisations (NGOs). The National Policy Framework and Strategy on Palliative Care (NPFSPC) aims to ensure adequate numbers of palliative care trained healthcare workers. Guidelines and tools such as the Supportive and Palliative Care Indicators Tool (SPICT) assist in identifying and caring for patients needing palliative care.

**Aim:**

To evaluate the knowledge, attitudes and practices of public sector doctors to provide palliative care in the Garden Route and Central Karoo Districts.

**Setting:**

The study was conducted at public sector district-level hospitals.

**Methods:**

A descriptive observational cross-sectional survey was conducted. The study population included all public sector district-level doctors. Participation was voluntary, and 73 responses (60%) were obtained. Data were collected with an online questionnaire using the adapted ‘Knowledge Attitudes Practice’ model. Quantitative data were imported into the Statistical Package for Social Sciences for analysis.

**Results:**

Participants had poor knowledge, attitudes and practices regarding palliative care. There was a statistically significant difference between the knowledge of junior doctors and senior doctors, with 78% of junior doctors having inadequate palliative care knowledge. Only 25% of respondents had received formal postgraduate palliative care training. Seventy (96%) participants reported that home was the best care setting for terminally ill patients.

**Conclusion:**

Doctors in the Garden Route and Central Karoo need further training to meet the NPFSPC standards.

**Contribution:**

This study adds to the palliative care field, highlighting the need for ongoing training of doctors.

## Introduction

Palliative care, as defined by the World Health Organization (WHO), is an approach that improves the quality of life of patients and their families who are facing problems associated with life-threatening illness.^1^ It prevents and relieves suffering through the early identification, correct assessment and treatment of pain and other problems, whether physical, psychosocial or spiritual.^1^ Globally, it is estimated that 40 million people have palliative care needs, of 78% (31 million) are in low- and middle-income countries (LMICs) such as South Africa. According to the WHO, only 14% of those requiring palliative care receive it.^1^ As per the 2014 World Health Assembly (WHA) Resolution 67.19 on Palliative Care, palliative care is an ethical responsibility of health systems and should be offered at all levels of care, and member states should develop policies to provide appropriate care.^2^

The need for palliative care in South Africa is evident from the Statistics South Africa 2018 report, where 59% of deaths in South Africa were from noncommunicable diseases (e.g. cancer, diabetes and heart disease), 29% from communicable diseases (e.g. human immunodeficiency virus [HIV], tuberculosis [TB]) and 12% from injuries.^3^

Based on the WHA Resolution and the need for palliative care in South Africa, the National Policy Framework and Strategy on Palliative Care 2017–2022 (NPFSPC) was developed in 2017. The NPFSPC estimates that 50% of patients who die in South Africa would benefit from palliative care and that it should be available to all patients in need, at all levels of care.

However, health system challenges limit equitable access to palliative care. Barriers include inconsistent service delivery, lack of qualified healthcare workers, medicine shortages, limited access to information and insufficient finances.^4^

While tertiary-level palliative care is necessary, most patient care takes place in health districts as part of community-based or home-based care.^4,5^ Strengthening service delivery at this level should improve patient care. At the district level, a multidisciplinary team of healthcare workers is usually involved.^4^ This includes family physicians, medical officers, nurses, allied workers and community health workers. This team is tasked with identifying patients requiring palliative care as well as referring and treating patients. In South Africa, most home-based palliative care is provided by nongovernment organisations (NGOs) such as Hospice Palliative Care Association of South Africa (HPCA) and by professional nurses, caregivers and social workers.^5^

One goal of the NPFSPC is to ensure that an adequate number of appropriately trained healthcare workers can provide palliative care at all levels.^4^ Objectives to achieve this include providing in-service training of current health workers, nurse prescribing of medication, ensuring undergraduate training of healthcare workers and strengthening postgraduate learning.^4^ According to a 2022 survey of the current state of undergraduate palliative medicine programmes at South African universities, seven out of the eight universities have a dedicated palliative medicine teaching programme.^6^ Most programmes integrate palliative medicine into other clinical modules, such as family medicine.^6^ However, combined teaching hours across the degree programmes are insufficient. Only one university met the recommended 40-h target by the European Association of Palliative Care, to attain undergraduate competency.^6^ Postgraduate palliative medicine training is offered by the University of Cape Town (UCT) in the form of a 1-year postgraduate diploma, 2-year Master’s degree in Philosophy (MPhil) in palliative medicine and a 10-week online introduction to palliative care short course.^5,7^ The Stellenbosch University offers a 12-week postgraduate online short course on palliative care in family medicine.^8^ Hospice Palliative Care Association offers additional training courses, including a 40-h introduction to palliative care course for professions.^9^ Palliative care training is advocated for healthcare workers with a lack of such training contributing to poor patient care.^10^ Practical themes highlighted for such training include pain and symptom management, use of syringe drivers and communication skills. Junior doctors often feel unprepared when dealing with patients needing palliative care, with regular requests for further training.^11^ In South Africa, healthcare workers also identified a lack of education on palliative care among medical staff for adolescent patients.^12^ A lack of professional education may be the highest barrier to physician’s morphine usage in clinical practice, and professional teaching could help patients report pain and effectively use the opioids prescribed.^13^

Clinicians’ attitudes towards palliative care have been noted to contribute towards poor clinical care.^10^ Many healthcare providers understand palliative care to be ‘care of the dying’ and only refer these patients late in the disease trajectory once active treatment has been exhausted.^10^ Misconceptions around palliative care exist in South Africa.^12^ These include that palliative care is only end-of-life care and that HIV is not an illness requiring palliative care.^12^ Practical misconceptions also exist on medication use, and physicians consistently report being concerned about high doses of opioids and the fear of side effects as barriers to adequate morphine prescribing.^13^ Doctors’ discomfort with palliative care leads them to prioritise active treatment and identify palliative needs late.^10^ The stigma of death surrounding palliative care has been noted among oncologists and haematologists, with associated negative emotions.^14^ Junior doctors have seen senior doctors disengaging from patients when end-of-life care is needed.^11^

Identifying patients needing palliative care has been noted to be a challenge, with a lack of referral criteria and limited palliative care resources contributing to this.^14^ In South Africa, healthcare system barriers to good palliative care have been noted.^12^ For example, young patients are missed in the transition to adult services.^12^ The absence of policies also means that palliative care is not consistently provided at facilities.^4^ Providers easily identify cancer patients as having palliative care needs, but other chronic illnesses can be missed.^10^ Advanced HIV disease is an example of this.^12^ For this reason, tools have been developed to assist healthcare workers to identify patients needing palliative care. An example of this is the Supportive and Palliative Care Indicators Tool (SPICT) and the SPICT-SA adapted specifically for South Africa.^15^ Practically, while other tools exist, it would be useful to get doctors to at least use this one tool.

Providing palliative care to patients involves multiple factors. Clinicians need theoretical knowledge, appropriate attitudes, practical skills and awareness of palliative care services. Training of healthcare workers, including district-level doctors, needs to target competency gaps in these areas. Research on the status of palliative care services in South Africa exists.^5,16^ In the George subdistrict of the Western Cape, the regional hospital has established an integrated palliative care programme.^17,18^ Patients are identified and referred from the regional hospital, tuberculosis hospital or primary healthcare clinics to the palliative care multidisciplinary group. The palliative care services this programme offers include weekly multidisciplinary team meetings, regular ward rounds, home visits, telephonic consultations and informal or formal training.^17^ However, no studies could be found that researched the knowledge, attitudes and practices among doctors working with patients needing palliative care in South Africa or in the Western Cape. Assessing these factors could provide valuable information on areas to focus to improve competencies of doctors providing palliative care in line with the NPFSPC standards.

### Aim and objectives

The aim of the study was to evaluate the knowledge, attitudes and practices of public sector doctors who provide palliative care to patients in the Garden Route and Central Karoo districts of South Africa.

Specific objectives included to assess doctors’ knowledge of palliative care and palliative care services, to determine doctors’ attitudes towards palliative care and to evaluate doctors’ practices of palliative care.

## Research methods and design

### Study design

This was a descriptive observational cross-sectional survey.

### Setting

The study was conducted at public sector district-level hospitals in the Garden Route and Central Karoo districts in the Western Cape Province of South Africa. There are seven district hospitals in the Garden Route and four in the Central Karoo, namely, Knysna, Mossel Bay, Oudtshoorn, Uniondale, Ladismith, Riversdale, Harry Comay Tuberculosis Hospital, Beaufort West, Laingsburg, Murraysburg and Prince Albert. Hospitals differed in numbers of doctors working per facility, depending on the size of the facility. All district hospitals employed medical officers (community service, sessional or part time or full time) and some employed interns, family medicine registrars and family physicians. Doctors’ responsibilities extended from the relevant hospital to the primary care clinics in the community.

The communities served were lower- and middle-income people of predominantly Afrikaans, isiXhosa or English home languages. The population of the Garden Route District was 621 245 people in 2020 with a Gini coefficient of 0.61 and an unemployment rate of 16%.^19^ The population of the Central Karoo was 74 763 in 2020 with a Gini coefficient of 0.59 and unemployment rate of 22%.^20^ The top five causes of life lost in the Garden Route and Central Karoo for 2015 were HIV/AIDS and TB, cancers, other noncommunicable diseases, cardiovascular disease and intentional injury.^21^ Palliative care was primarily initiated and continued at the primary care and district hospital level by doctors and other healthcare workers. Additional services varied per subdistrict and may have included nonprofit organisations, such as HPCA. George Regional Hospital is the referral hospital for both districts, providing the next level of care through specialist departments to all district hospitals, including a family medicine department. The Family Medicine Department at George Hospital conducted outreach site visits to each of the district hospitals mentioned to provide training and support to registrars in the family medicine programme.

### Study population

The study population was all doctors (interns, community service medical officers, sessional medical officers, full-time medical officers, registrars and family physicians) working at the aforementioned district-level hospitals in the Garden Route and Central Karoo. Locum doctors and outreach doctors (specialists, registrars or medical officers) from George Regional Hospital were excluded as they work above district-level in the regional setting.

### Sampling

The study population calculated from the recent 2022 employment records from all facilities was 121. Using a 95% confidence interval (CI) and 5% error margin, a sample size of 93 was targeted (response rate 75%). To achieve this number, the entire study population was invited, and no sampling was used. Doctors were recruited and encouraged to participate through a designated liaison per facility assisted by the George Family Medicine outreach team. Following voluntary informed consent, the final number of participants in the study was 73, with a response rate of 60%.

### Pilot

A pilot study was conducted using two voluntary family medicine registrars from facilities outside of those included in the study. Participants were requested to complete the questionnaire through the same methods as for the actual study and asked to provide feedback. The feedback and results were considered before distributing the final questionnaire. No changes were made to the tool.

### Data collection

Data collection occurred over 5 months in the first half of 2023. A questionnaire was created based on the Knowledge, Attitudes and Practice (KAP) model.^22^ The KAP survey design is used to gain information around what is known (knowledge), believed (attitudes) and done (practiced) regarding the chosen topic.^23^ The questionnaire was adapted from a combination of questions from three previously validated tools from studies conducted in Kuwait, China and Hungary.^24,25,26^ The questionnaire assessed how doctors identify patients, familiarity of services available and knowledge of theoretical as well as practical aspects of palliative care, such as prescribing medications. Doctors’ attitudes towards the concept of palliative care and early referral of patients were determined. The last component quantified doctors’ practice of palliative care in their facilities, looking at practicalities such as medications used, resources available and the utilisation of tools (specifically the SPICT). The questionnaire included multiple choice questions (including true, false or don’t know questions), multiple response questions and Likert scales.

The knowledge section of the questionnaire consisted of two types of questions, with a total of 14 questions. A 5-point Likert score was used for three questions where participants assessed their self-knowledge in pain management, other symptom management and counselling. Five points were given for excellent, four for good, three for fair, two for poor and one for none for each of these questions. The second set of questions consisted of 11 multiple-choice questions for there was one correct answer for each question. One point was given for each correct answer and zero for every incorrect answer. For each participant, a total knowledge score was calculated as the sum of all knowledge points, with a maximum score of 26. Poor knowledge was calculated as a score of less than 50% (13 out of 26), fair knowledge of between 50% and 74% and good or adequate knowledge as a score of above 75%.

There were 11 questions that determined the attitudes of participants towards palliative care. The last seven questions of the questionnaire focused on practices of palliative care.

The questionnaire was validated by means of an expert panel of five palliative care educated professionals, research experts and experts in local health facilities. Experts assessed all questions in terms of relevance to the research question and whether they were formulated correctly. The questionnaire was adapted based on their recommendations and converted to an online format using REDCap software. The invitation link to the questionnaire was distributed first via email to a designated liaison for each facility and then to participants via WhatsApp messenger from the liaison person. Outreaching family physicians from George Regional Hospital assisted by encouraging participation on their outreach trips to the included facilities. Three reminders to complete the questionnaire were sent via WhatsApp messenger through the liaison at each facility.

### Data analysis

Quantitative data from the survey software (REDCap) were exported and then imported into SPSS v23 (Statistical Package for Social Sciences) for analysis. The data were analysed by the primary investigator with assistance from a statistician from Stellenbosch University and research supervisors.

Data were analysed using descriptive statistics. Categorical data were presented as percentages and frequencies.

Results were compared from two groups of doctors – namely, junior doctors (interns, community service medical officers, grade 1 medical officers and junior registrars) and senior doctors (grade 2 and 3 medical officers, senior registrars and specialists). Categorical variables were compared using the chi-square test and a choice of statistical tests was made with the statistician and supervisor.

### Ethical considerations

Ethics approval for the study was obtained from the Human Research Ethics Committee at Stellenbosch University (Reference number: S22/05/089). Permissions were granted from the Western Cape Government (Reference number: WC_202209_011). The questionnaire was voluntary, anonymous and completion implied consent.

## Results

### Demographics

A total of 73 participants were included: 46 junior doctors and 27 senior doctors. The breakdown of rank of medical doctor and place of work is demonstrated in Table 1 and Table 2.

**TABLE 1:** Rank of participants (*N* = 73).

Rank	Frequency	%
Medical intern	5.0	6.85
Community service medical officer	23.0	31.51
Grade 1 medical officer	16.0	21.92
Grade 2 medical officer	8.0	10.96
Grade 3 medical officer	8.0	10.96
Family medicine junior registrar	2.0	2.74
Family medicine senior registrar	2.0	2.74
Family physician	9.0	12.33
**TOTAL**	**73.0**	**100.00**

**TABLE 2:** Workplace of participants (*N* = 73).

Workplace	Frequency	%
Knysna Hospital	21.0	28.77
Mosselbay Hospital	12.0	16.44
Oudtshoorn Hospital	8.0	10.96
Uniondale Hospital	3.0	4.11
Ladismith Hospital	1.0	1.37
Riversdale Hospital	4.0	5.48
Harry Comay TB Hospital	4.0	5.48
Beaufort West Hospital	13.0	17.81
Murraysburg Hospital	2.0	2.74
Prince Albert Hospital	2.0	2.74
Laingsburg Hospital	3.0	4.11
**TOTAL**	**73.0**	**100.00**

Among the participants, 55 (75%) had received no formal training in palliative care (diploma, 5-day course, other course, postgraduate training) while 18 (25%) had received formal training.

### Knowledge

**FIGURE 1:**
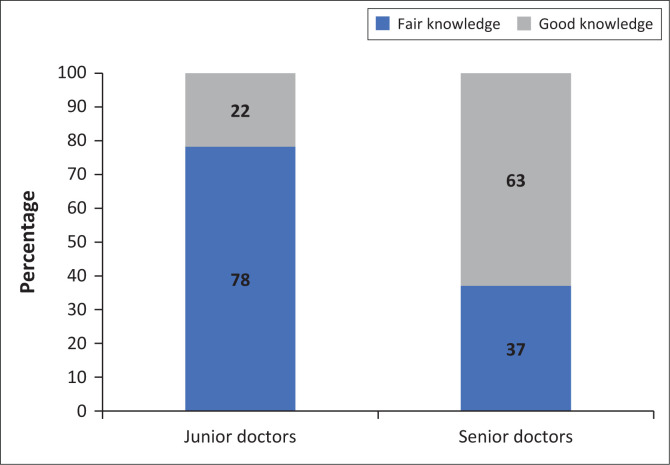
Comparison of knowledge between junior and senior doctors.

Forty-six (63%) participants had a fair knowledge (CI 51.2–73.4), while 27 (37%) had good or adequate knowledge (CI 26.6–48.8). A comparison was made between the knowledge scores of junior doctors and senior doctors using the chi-square test. There was a statistically significant difference between the two groups (*P* 0.001), with 63% of senior doctors having good knowledge while only 22% of junior doctors had good knowledge (see Figure 1).

Sixty-four (88%) participants correctly identified that palliation was not the same as hospice care, 58 (81%) participants reported that palliative care should be provided for patients for whom no curative treatments were available and 67 (93%) identified that palliative care should be provided alongside anticancer treatment. In terms of practical knowledge, 15 (21%) participants did not know what an advance directive was, and 72 (99%) participants did know what a ‘do not resuscitate’ order was.

### Attitudes

**FIGURE 2:**
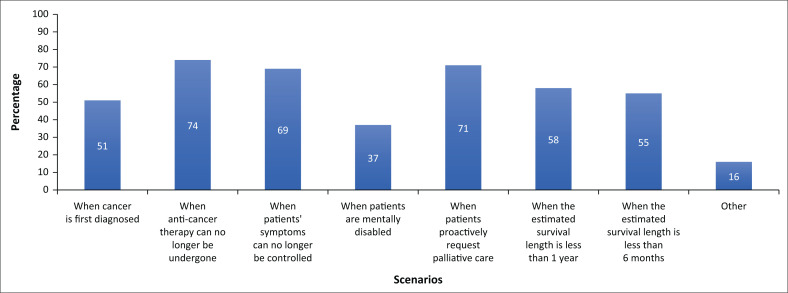
Scenarios selected by participants when asked when cancer patients should receive palliative care.

Forty-six (63%) participants said they would recommend palliative care to cancer patients who attended the clinic for the first time, 16 (22%) would not and 11 (15%) did not know. See Figure 2 for additional scenarios selected by participants describing when cancer patients should receive palliative care. Forty (55%) participants said they believed palliative care can improve patients’ survival and 73 (100%) said that early palliative care can improve patients’ quality of life. Forty-seven (64%) participants believed that they should inform patients of an unfavourable prognosis, 13 (18%) said it depended on the patients’ wishes and 13 (18%) said it depended on the situation. Seventy-one (97%) participants said they should follow a patients’ wishes when they prefer to forgo life-sustaining treatments. Thirty-eight (52%) participants did not approve performing cardiopulmonary resuscitation (CPR) on terminally ill patients, while 35 (48%) said it depended on the situation. Forty-three (58%) participants said they would support the patient’s wishes, should conflict arise between the patient and the family in the decision-making process.

If a patient was no longer competent and the family’s wishes conflicted with those previously expressed by the patient, 36 (49%) said they would support the patient while 35 (48%) said it depended on the situation. Seventy (96%) participants said that home was the best care setting for terminally ill patients, 39 (53%) said a nursing home and 17 (23%) said hospital (participants could select more than one option). The final question asked participants what they believed to be the most appropriate type of care for terminally ill patients. No participants selected continuous curative treatment of the disease until death, 39 (53%) selected a combination of curative treatment and palliative care, while 33 (45%) selected palliative care only.

### Practices/facilities

**FIGURE 3:**
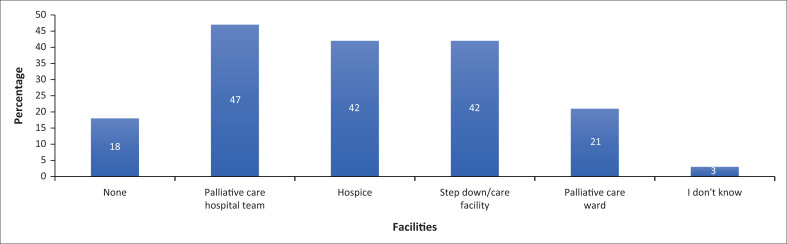
Palliative care facilities available in participant settings.

Forty-eight (66%) participants said that healthcare services in their region were currently available for the care of palliative care patients, 20 (27%) said that services were not available and 5 (7%) did not know what services were available. Figure 3 demonstrates the types of facilities available. A 5-point Likert scale was used to assess whether participants thought families of terminally ill patients could provide appropriate care in the home. Three (4%) strongly agreed, 26 (36%) agreed, 9 (12%) were unsure, 32 (44%) disagreed and 6 (8%) strongly disagreed. Regarding participants’ ability to set up a syringe driver, 3 (4%) participants said they had no ability, 23 (32%) poor ability, 23 (32%) fair ability, 18 (25%) good ability and 6 (8%) had an excellent ability.

Fifty-one (70%) participants were not familiar with the SPICT, and 61 (84%) said they had never used the SPICT. The final question asked participants medications were available in their settings. Sixty-eight (93%) said morphine syrup was available, 67 (92%) said lorazepam, 67 (92%) said diazepam, 68 (93%) said haloperidol, 71 (97%) said metoclopramide, 25 (34%) said ondansetron and 56 (77%) said hyoscine butylbromide.

## Discussion

Senior doctors had better overall knowledge than the junior doctors. The NPFSPC aims to ensure adequate numbers of appropriately trained healthcare workers who can provide palliative care at all levels, with undergraduate training as one of their objectives to achieve this.^3^ Even though most South African universities offer palliative medicine integrated into their undergraduate programmes, knowledge from the junior group in the study was inadequate with three quarters scoring below 74%. Among participants who responded ‘poor’ to the ‘experience’-related questions specifically, the majority came from the junior group. This alludes to the fact that junior doctors lack clinical experience when it comes to managing palliative care patients. However, as these were self-assessment questions, doctors may have scored themselves lower because of modesty or poor self-confidence. Perhaps the curriculum content of South African undergraduate programmes should be reviewed in more depth to improve junior doctors’ knowledge of palliative care and boost appropriate confidence. Additionally, there is a need to provide postgraduate training to both junior and senior doctors, as only a quarter of the entire study group had received formal palliative care training.

The WHO definition of palliative care emphasises the need for early identification of palliative care problems to prevent suffering and improve quality of life.^1^ However, only half of participants said they would recommend palliative care to cancer patients at the time of first consultation even though all agreed that palliative care improves quality of life. This is, however, a higher number than a Chinese study where only a fifth of participants would refer patients at first consultation.^25^ There were conflicting opinions surrounding end-of-life care, with almost the whole group reporting they would follow a patient’s wishes to forgo life-sustaining treatment, yet only half reporting they would support the patient, should conflict arise between the patient and the family – whether the patient was competent or incompetent to make decisions. Respecting a patient’s autonomy in decision making was therefore not prioritised by the whole group. This can be linked to the knowledge question where one-fifth of participants did not know what an advance directive was. In the United States, clinicians’ attitudes favoured honest and accurate disclosure to patients while Hungarians used their own judgement to decide on the information provided to patients or families. This study showed that attitudes towards patient autonomy were more like those of the Hungarian study. The Hungarian setting is similar to the Garden Route where access to palliative care knowledge and facilities is limited.

Almost half the group did not approve performing CPR on terminally ill patients, while the rest said it depends on the situation. Participants identified the home as being the best care setting for terminally ill patients, with almost all selecting this option, followed by half selecting nursing home and one quarter selecting hospital. However, when asked whether the families of terminally ill patients could provide appropriate care in the home, half disagreed. This identified another conflicting opinion, where doctors wanted to send palliative patients’ home for care but do not feel the families could provide that care. This could be influencing doctors’ decisions to discharge patients into family care and is an area that interventions can be focused on. The same was true in the Hungarian study where home-based care was limited, and terminal patients were admitted to hospital for end-of-life care.^26^ Both the Hungarian and Kuwait studies showed a positive association between knowledge and attitudes, whereby better knowledge tended to lead to more positive attitudes towards palliative care.^21,26^ Hence, the focus should be on providing training to improve the knowledge gaps identified in this study, and consequently, attitudes and hopefully practices should improve.

Over a third of participants said their ability to set up a syringe driver was none to poor and another third said fair. This is an easy skill that can be performed by junior or senior doctors. Providing training on this specific skill is achievable and will improve end-of-life care to patients.

Certain patients have an increased need for palliative care, regardless of prognosis.^4^ To help identify these patients, validated tools have been created. One such example is the SPICT.^15^ A minority of participants were familiar with the SPICT and most said they had never used it. Improving awareness about the availability of these tools will increase the number of appropriate patients identified by doctors and thus offered palliative care.

Awareness on the availability of palliative care services and facilities was low. Less than half of the participants reported that doctors had access to a palliative care hospital team, hospice or a stepdown ward, while only a fifth had access to a dedicated palliative care ward in order to provide care for patients. Some participants reported no access to any palliative service or facility. The lack of inpatient facilities for palliative patients in this study setting does not meet the NPFSPC standards. This emphasises the need for improved palliative care services at the primary healthcare level and in the homes of patients. Educating district-level doctors will empower them to initiate services such as in-hospital or community-based teams that do not necessarily require physical infrastructure.

In terms of drugs used in palliative care, morphine syrup, lorazepam, diazepam, haloperidol and metoclopramide were reported as readily available in the study setting while hyoscine butyl bromide was less readily available and ondansetron was only available in a third of settings. It was reassuring to know that there were drugs available for doctors to use, even if they required training on how best to do so.

### Strengths and limitations

This study is the first of its kind in the Western Cape and in Southern Africa. It can therefore be used as a starting point to guide training goals to meet NPFSPC targets. The strengths include representation from all district hospitals, included both junior and senior doctors and assessed a variety of important indicators relevant to care in a district setting in South Africa.

The total population of doctors was 121. Using a 95% CI and 5% error margin, a sample size of 93 was targeted with a 75% response rate. However, the final number of participants in the study was 73 with a response rate of 60%. This limited the degree of statistical analysis that could be done. Not all the questions were answered by all the participants. Ten questions had one participant who did not answer, and two questions had two participants who did not answer. However, this was not a large number to influence final data.

## Conclusion

Postgraduate training to both junior and senior doctors is needed to reach the NPFSPC goals. Training should focus on specific gaps identified in this study, namely, education on end-of-life concepts such as advance directives and patient autonomy, practical skills such as setting up syringe drivers and the use of readily available palliative care drugs, including morphine. Although now included in most undergraduate curricula, there may be a need to review the contents of palliative medicine in undergraduate programmes at South African universities to improve knowledge of palliative care among junior doctors. Awareness of the SPICT-SA specifically was inadequate, and educating doctors about the availability of this tool should increase the number of patients correctly identified and offered early palliative care. Doctors’ reported access to palliative care services was low; therefore, empowering doctors through further training and awareness of palliative care services could strengthen district-level palliative care through the development of inpatient and community-based palliative care teams.
